# Prevalence of vocal handicap among primary school teachers in Malaysia and validation of a noise-measuring wearable device to measure vocal intensity

**DOI:** 10.7717/peerj.21522

**Published:** 2026-08-13

**Authors:** Zainal Shaffiq Ahmad Shukkeri, Sheng Qian Yew, Marina Mat Baki, Nor Shahrina Mohd Zawawi

**Affiliations:** 1 Department of Public Health Medicine, Faculty of Medicine, Universiti Kebangsaan Malaysia, Bandar Tun Razak, Cheras, Kuala Lumpur, Malaysia; 2 Department of Otorhinolaryngology, Faculty of Medicine, Universiti Kebangsaan Malaysia, Bandar Tun Razak, Cheras, Kuala Lumpur, Malaysia; 3 Speech Therapy Unit, Hospital Canselor Tuanku Muhriz, Bandar Tun Razak, Cheras, Kuala Lumpur, Malaysia

**Keywords:** School teachers, Voice disorders, Wearable electronic devices

## Abstract

**Introduction:**

The prevalence of vocal handicap among primary school teachers in Malaysia has not been previously investigated, and effective preventive strategies remain lacking. This study aimed to determine the prevalence of vocal handicap among primary school teachers in Negeri Sembilan, Malaysia, and to evaluate the validity and reliability of a wearable device, originally designed for noise measurement, for assessing vocal intensity in this population.

**Materials & Methods:**

The study was conducted in two phases. Phase 1 was a cross-sectional survey using the Malay-Vocal Handicap Index-10 (M-VHI-10) to assess the prevalence of vocal handicap among primary school teachers in Negeri Sembilan, Malaysia. Phase 2 was a cross-sectional validation study in which voice intensity measured by the wearable device was compared with that of a voice dosimeter (gold standard); concurrent validity by Pearson correlation; agreement by intraclass correlation (ICC [3,1]).

**Results:**

A total of 132 (response rate = 92.3%) and 28 (response rate = 100.0%) teachers participated in Phase 1 and Phase 2, respectively. The overall prevalence of vocal handicap was 79.5%, with teachers using voice amplifiers showing a significantly higher prevalence (89.8%) compared to those not using voice amplifiers (73.5%) (*p* = 0.025). No significant differences were observed between different school types (*p* = 0.105). The wearable device exhibited poor concurrent validity (*r* = 0.184, *p* = 0.330) and poor inter-rater reliability (ICC = 0.077, *p* = 0.171) in measuring vocal intensity compared with a voice dosimeter (gold standard).

**Conclusion:**

The prevalence of vocal handicap among primary school teachers in Negeri Sembilan, Malaysia, was higher than that reported in other countries. Although it was initially intended to serve as a surrogate instrument for assessing vocal intensity, the wearable device, originally developed for noise measurement, exhibited poor validity and reliability. These findings highlight the need for the development of a novel device specifically designed to measure vocal intensity accurately.

## Introduction

Continuous vocal use is not only common among school teachers, but it can also increase their vocal load and subsequently lead to negative and profound impacts in this working population (Feng et al., 2022). Increased vocal load is defined as an increase in phonatory effort or phonation at higher frequency, intensity, and/or duration compared with normal conditions (Hunter et al., 2020). When identified early, lifestyle adjustments and better voice care can often reduce its negative impact and prevent progression to more serious voice problems. However, if this issue is not addressed early, increased vocal load may lead to vocal fatigue as well as vocal disorders such as vocal nodules (Won et al., 2016) and vocal polyps (Joseph, Joseph & Jacob, 2020). Finally, when these voice problems or voice impairments affect an individual’s daily life, a condition known as vocal handicap will occur (Moghtader et al., 2020).

It is widely known that school teachers rely heavily on their voices as the primary tool for communication in the classroom (Castillo-Allendes et al., 2024). Therefore, vocal handicap can reduce and compromise teaching effectiveness (Moreno, Calvache & Cantor-Cutiva, 2025). As a result, this may lead to decreased comprehension, engagement, and overall learning outcomes, ultimately diminishing the effectiveness of learning objectives (Moy et al., 2015). Secondly, the physical discomfort and pain associated with vocal handicaps not only disrupt the teaching process but also interfere with teachers’ ability to concentrate on their students (Hunter et al., 2020). This can disrupt classroom interactions, thereby making communication between teachers, students, and colleagues more difficult. In addition, the psychological impact of vocal handicap is profound (Shoeib, Nassar & Ghandour, 2012). It can lead to increased stress (Shoeib, Nassar & Ghandour, 2012), anxiety (Shoeib, Nassar & Ghandour, 2012), and emotional disturbance (Adamu et al., 2022) among teachers. Furthermore, vocal handicap may limit career opportunities by causing sickness absenteeism (Moy et al., 2015). Even more concerning, vocal handicaps can affect teachers’ personal lives. The physical and emotional strain resulting from vocal handicap can disrupt personal relationships and lead to feelings of isolation (Adamu et al., 2022). Teachers may be unable to participate in social activities or hobbies that require communication, thereby negatively affecting their overall quality of life (Almuzaini, 2025).

A meta-analysis consisting of 62 studies reported a pooled prevalence of vocal disorders among teachers of 37.7% (Baghban, Golmohammadi & Asadollahpour, 2025). However, this prevalence data was not specific to vocal handicap alone. Studies have shown that the prevalence of vocal handicap among teachers ranges from 10.4% to 55.5% (Moy et al., 2015; Sampaio et al., 2012; Melo et al., 2023; Alharbi et al., 2024; González et al., 2022). Nonetheless, these data were mostly reported from South America. The prevalence of vocal handicap varies from country to country and from one school to another, as it is influenced by multiple factors within the teaching profession, such as speaking duration, noisy environments, opportunities for vocal rest, training in voice use techniques, workload, emotional demands, and varying levels of work-related stress (Phyland & Miles, 2019). Despite a local study reporting a 10.4% prevalence of vocal handicap among secondary school teachers (Moy et al., 2015), this figure does not reflect the prevalence among primary school teachers, who typically need to exert greater vocal effort when teaching and managing young children. Hence, there is a need to determine the prevalence of vocal handicap among primary school teachers in Malaysia and an approach or tool to effectively prevent vocal handicap.

One effective evidence-based, non-surgical approach to alleviating vocal handicap is vocal rest (Fujiki, Huber & Sivasankar, 2021). In other words, preventing vocal handicap relies on minimising vocal exertion from the very beginning. However, achieving complete vocal rest in a school environment is particularly challenging. This is because, in primary schools, classrooms are often noisy, students’ behaviour can be difficult to manage, and teachers frequently need to raise their voices to be heard. Secondly, behavioural interventions such as semi-occluded vocal tract (SOVT) exercises (*i.e.,* voice exercises where the mouth or lips are partially closed during phonation) (Heller-Stark et al., 2024) and vocal hygiene training (Sundram et al., 2019) are also effective in preventing vocal handicap. Nonetheless, their real-world implementation among teachers may be limited by the lack of proper training from professionals in speech-language pathology, time constraints within busy classroom settings, and limited awareness and motivation.

To achieve vocal handicap prevention while overcoming the above limitations, one potentially pragmatic tool is wearable technology. Wearable devices, also known as wearable technologies, encompass electronic and digital devices integrated into clothing, accessories, and other items that can be comfortably worn on the human body (Singhal & Chopra, 2021). Currently, wearable technology has been applied across various healthcare fields, including acoustics (Kong et al., 2024), gastrointestinal disease management (Reddy & Chawla, 2025), cardiology (Hughes et al., 2023), and respiratory medicine (Aliverti, 2017). It is anticipated that wearable devices will be able to accurately measure vocal intensity and eventually prevent vocal handicap. In essence, the tool should be capable of monitoring vocal intensity in real time, providing instantaneous alerts when vocal intensity exceeds safe vocal load thresholds, functioning reliably in noisy environments such as classrooms, and being freely accessible online.

Based on the literature, the voice dosimeter is considered the gold standard for measuring vocal intensity (Llorente et al., 2024; Misono et al., 2015; Titze, Švec & Popolo, 2003). Nonetheless, it also has various limitations. For instance, it does not provide instantaneous alerts when vocal load thresholds are exceeded and does not offer real-time vocal intensity readings. Instead, the recorded data must be processed and analysed offline using Praat software. Not to mention, the cost of a voice dosimeter is relatively high, typically ranging from USD 500 to USD 5,000 (Llorente et al., 2024). In the absence of a practical, portable, and cost-effective tool for measuring vocal intensity with real-time feedback, and given financial constraints, the research team (comprising an otolaryngologist, a speech therapist, and an occupational health doctor) therefore explored existing mobile applications originally designed for noise measurement. It was hoped that such applications could be adapted, using additional hardware, for voice monitoring. The selected applications were required to meet four criteria: (i) reliable performance in noisy environments, (ii) provision of real-time measurements, (iii) ability to deliver immediate feedback or alerts when a predefined threshold is exceeded, and (iv) free availability on either the Apple App Store or Google Play Store.

In essence, this study aimed to determine the prevalence of vocal handicap among primary school teachers in Negeri Sembilan, Malaysia, and to evaluate the validity and reliability of a wearable device for measuring vocal intensity. In terms of validation, it was hypothesised that Pearson’s correlation and the intraclass correlation coefficient (ICC) would be greater than 0.50, respectively.

## Materials & Methods

This study consisted of two phases. Phase 1 aimed to investigate the prevalence of vocal handicap among primary school teachers in Negeri Sembilan, Malaysia. Subsequently, Phase 2 aimed to determine the validity and reliability of a wearable device in measuring vocal intensity among primary school teachers in Negeri Sembilan, Malaysia.

### Phase 1

**Figure 1:**
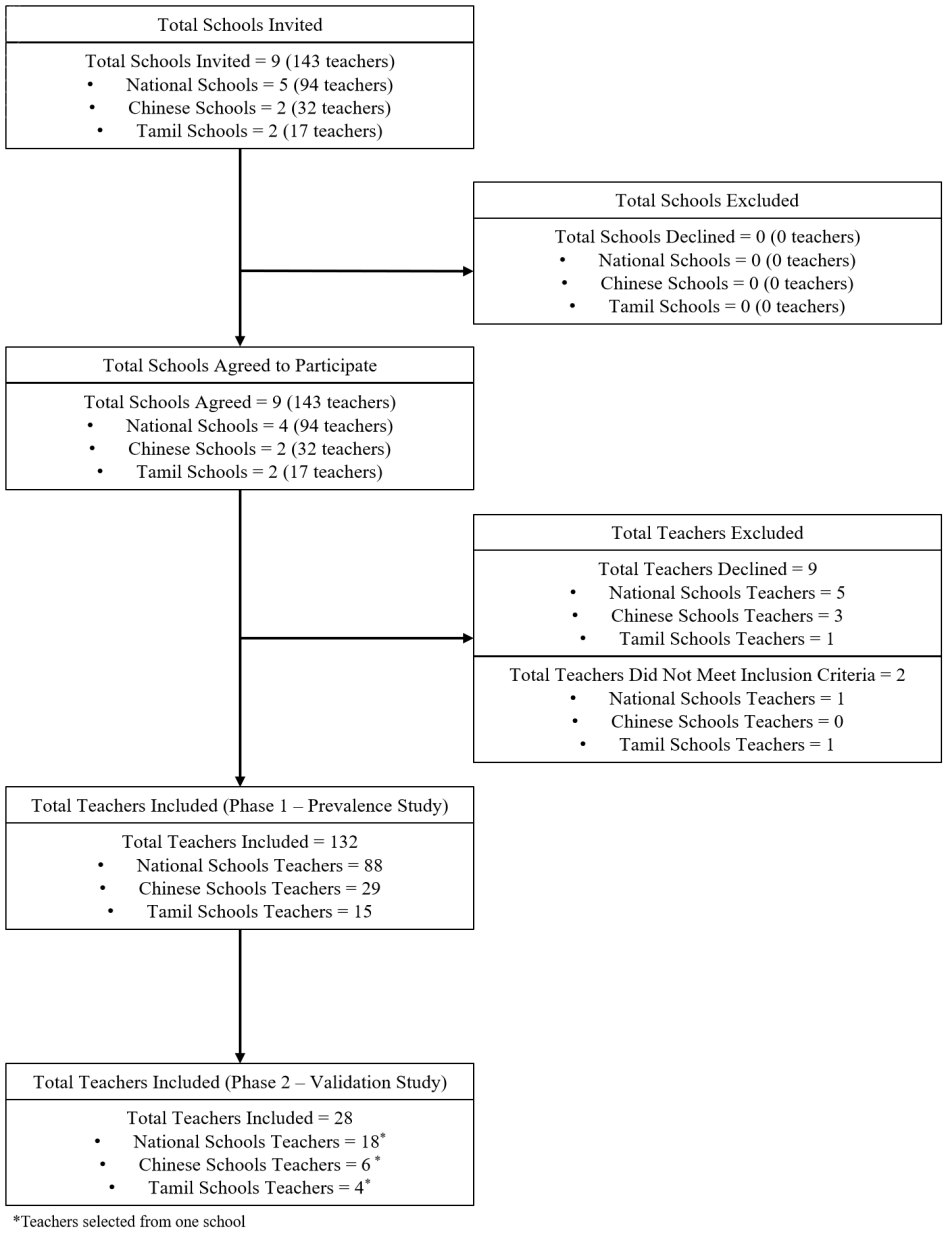
Study flowchart of the present study.

Phase 1 was a cross-sectional study conducted from August to September 2025. This phase aimed to identify the prevalence of vocal handicap among primary school teachers in Negeri Sembilan, Malaysia. The study flowchart is illustrated in Fig. 1.

 To be included in the study, primary school teachers had to meet the following criteria: (i) employed at any public primary school in Negeri Sembilan, Malaysia; (ii) engaged in full-time teaching at a public primary school for at least one year; (iii) of any age, gender, or ethnicity; and (iv) teaching any subject, including music and physical education. However, teachers with the following characteristics were excluded from participation: (i) currently experiencing any voice disorder during the study period; (ii) undergoing speech therapy at the time of participation; (iii) on extended leave during the study period (such as maternity leave or study leave); and (iv) involved in private tutoring after regular school hours.

Given that the prevalence of vocal handicap was 10.4% among secondary school teachers in Malaysia (Moy et al., 2015), with a margin of error of 0.05, and a confidence interval of 95%, the required sample size was 143. This sample size was calculated using the Kish formula for infinite population (Kish, 1995).

Proportionate stratified sampling was employed in Phase 1 of the study, whereby schools in Negeri Sembilan were stratified into three types of schools, based on the language of instruction. According to the Negeri Sembilan State Education Department, there were 351 primary schools in the state (*i.e.,* 208 national primary schools, 82 Chinese primary schools, and 61 Tamil primary schools). In total, there were 9,567 teachers in the state (*i.e.,* 6,935 in national primary schools, 1,637 in Chinese primary schools, and 995 in Tamil primary schools). The number of schools and number of teachers in Negeri Sembilan, Malaysia were tabulated in Table 1 below.

**Table 1:** Types of school and the number of teachers in Negeri Sembilan, Malaysia.

**Types of school**	**No. of schools**	**No. of teachers**	**Proportion (%)**
National Primary School	208	6,935	72.5
Chinese Primary School	82	1,637	17.1
Tamil Primary School	61	995	10.4
Total	351	9,567	100.0

With the approval and assistance of the Director of the Negeri Sembilan State Education Department, official invitation letters were sent to the headmasters of all primary schools *via* the various district education offices. At the school level, a list of teachers’ names was obtained from the administrative office. This list served as the sampling frame. Subsequently, teachers (also referred to as sampling units) were randomly selected from each school. This process utilised an online random number generator.

During participant recruitment, all 143 selected teachers were provided with a Google Form *via* WhatsApp. The Google Form consisted of study information, a consent form, a checklist of inclusion and exclusion criteria, a sociodemographic factors survey form, and the Malay Version of the Voice Handicap Index-10 (M-VHI-10) questionnaire.

The M-VHI-10 was used to determine the prevalence of vocal handicap among primary school teachers. The VHI is a questionnaire comprising 10 items across three domains (*i.e.,* functional, physical, and emotional domains). Participants were required to respond using a five-point Likert scale (0 = never, 1 = almost never, 2 = sometimes, 3 = almost always, and 4 = always). Items 1 to 5 represent the functional domain, items 6, 7, and 10 represent the physical domain, and items 8 and 9 represent the emotional domain. In terms of score interpretation, items within each domain can be summed to yield a domain score. Alternatively, all 10 items can be summed to produce a total score ranging from 0 to 40. A total score of >11 points is considered to indicate vocal handicap (Ong et al., 2019). This cut-off point was adapted in this study, although a local study suggested a more stringent cut-off score of 7.5 points (Idris et al., 2025). Based on previous studies, the M-VHI-10 demonstrated good validity and reliability, with a Cronbach’s alpha of 0.77 and an intraclass correlation coefficient (ICC) ranging from 0.65 to 0.78 (Ong et al., 2019). In terms of validity, both total and domain scores were significantly higher in individuals with voice disorders (20.92 ± 8.74 points) compared with healthy volunteers (1.54 ± 1.97 points), indicating excellent discriminant validity (Ong et al., 2019).

Up to three reminders were sent to teachers who did not respond within a two-week period. Teachers who failed to respond after the third reminder were considered non-respondents. To minimise missing data, the Google Form was configured to require participants to answer all questions before submission. To prevent duplicate responses from the same participant, the researchers enabled the “one response only” setting so that each participant could take part only once.

For descriptive analysis, categorical variables were presented as frequencies and percentages. Continuous variables were presented as means with standard deviations or medians with interquartile ranges, depending on data normality. The Kolmogorov–Smirnov test was used to assess data normality. The prevalence of vocal handicap was expressed as a percentage. All statistical analyses were conducted using SPSS Version 28.

### Phase 2

Phase 2 of the study aimed to determine the validity and reliability of a wearable device for measuring vocal intensity among primary school teachers in Negeri Sembilan, Malaysia. A cross-sectional study was scheduled from October to November 2025. This phase involved three primary schools (*i.e.,* one national primary school, one Chinese primary school, and one Tamil primary school) located in Negeri Sembilan, Malaysia. This approach was intended to ensure adequate representation of teachers from each type of school.

Since the validation of the wearable device was determined using concurrent validity testing and inter-rater reliability testing, the sample size for Phase 2 was calculated using the Walter formula (Walter, Eliasziw & Donner, 1998). Based on expert opinion, the acceptable reliability (R_0_) = 0.50, expected reliability (R_1_) = 0.80, significance level = 0.05, power of the study = 80%, and number of raters (k) = 2. Hence, the required sample size was 28. For sample size estimation using concurrent validity, assuming significance level = 0.05, power = 80%, a sample size of 29 would be required to detect a moderate correlation (*r* ≥ 0.50).

Hence, a total of 28 teachers (*i.e.,* 14 with vocal handicap and 14 without vocal handicap) from Phase 1 were purposely sampled to participate in Phase 2. Their participation was subject to the same inclusion and exclusion criteria outlined in Phase 1. These teachers received an information booklet detailing the objectives and procedures of the study, along with a consent form. Upon receipt of the signed informed consent form, the researchers provided a comprehensive explanation of the study procedures to the respective teachers. Appreciation was expressed to teachers who declined participation or did not meet the inclusion criteria, and they were excluded from the study.

**Figure 2:**
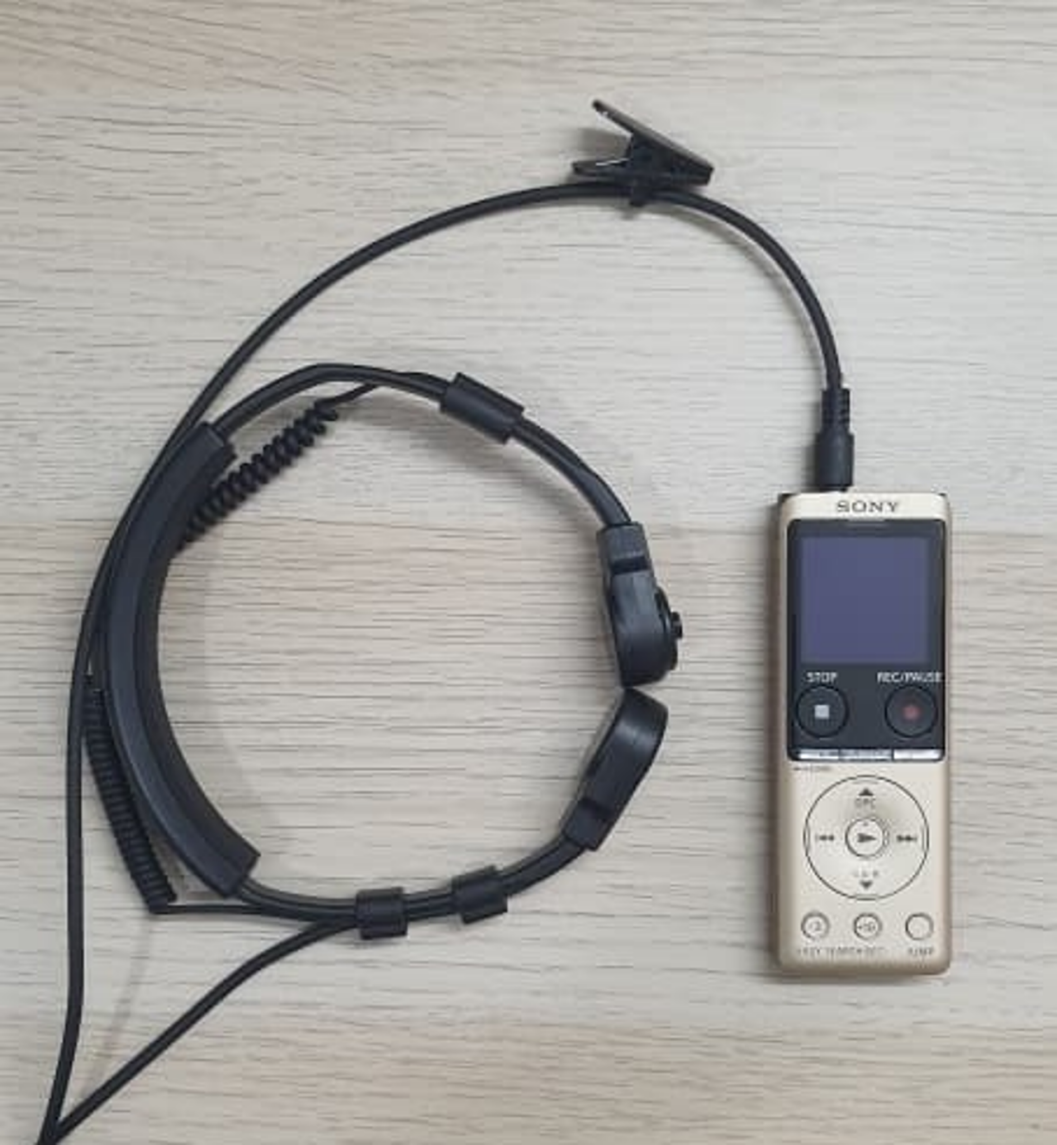
The setting of a voice dosimeter.

The validity and reliability of the wearable device in measuring vocal intensity were assessed against a voice dosimeter (Fig. 2), which was the gold standard. The voice dosimeter used in this study was in accordance with the protocol described by Bottalico & Nudelman (2023), operating with an air-conduction microphone. The device recorded sound pressure levels at a sampling rate of 44.1 kHz, using A-weighting and a fast time constant for signal processing. Calibration was performed using a sound calibrator at 94 dB and 1 kHz, in accordance with standard acoustic calibration procedures.

**Figure 3:**
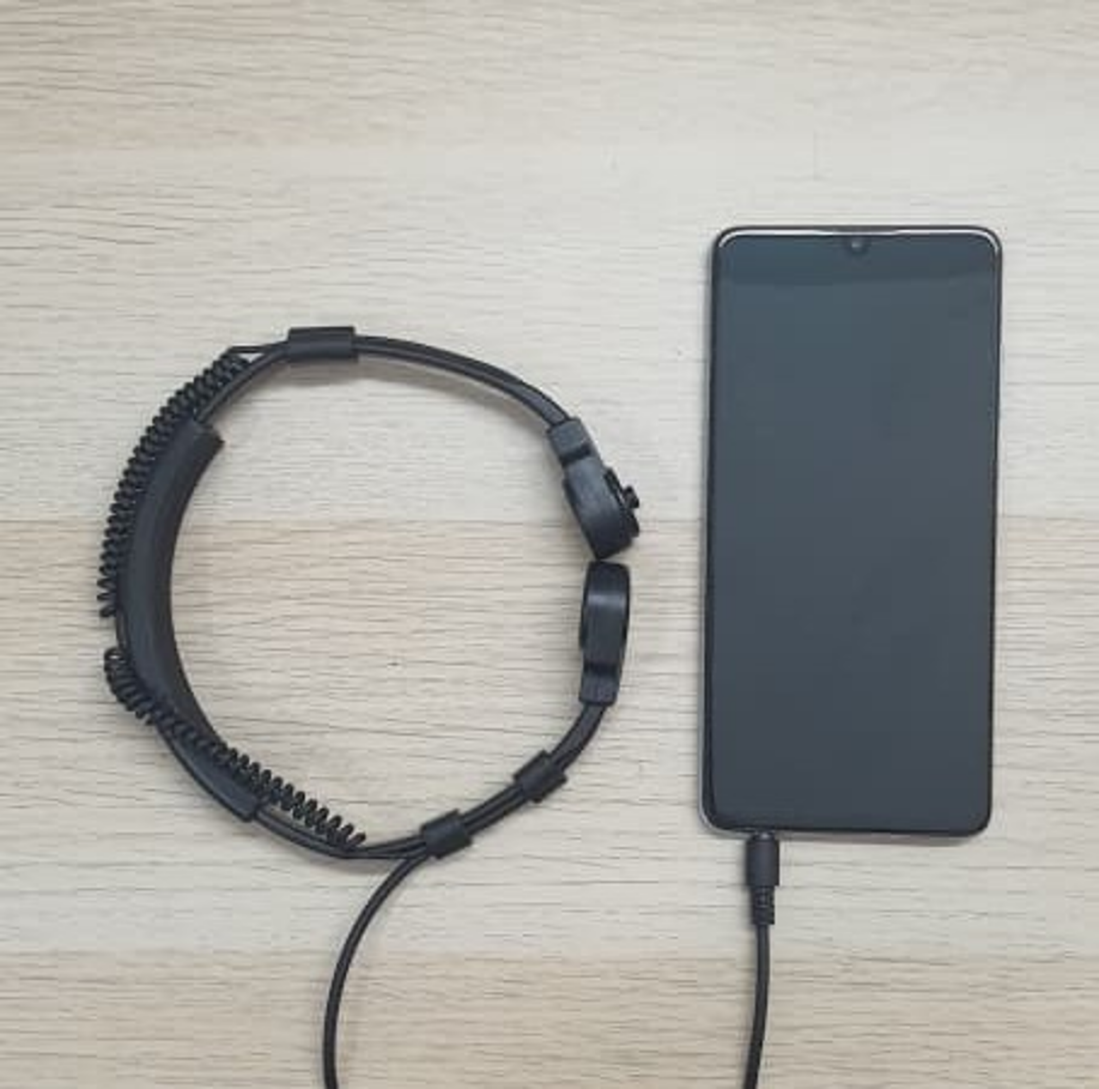
A wearable device.

The wearable device comprised a collar microphone connected to a smartphone (Fig. 3). A mobile application, Sound Meter (version 2.13.4), was downloaded from the Google Play Store and installed on the smartphone. This application was chosen based on the four criteria outlined in the introduction section. Firstly, the Sound Meter application measures sound pressure levels in real time in decibels using A-weighting and a slow time constant. The equivalent continuous sound level (L_eq_) was used for the sound pressure level measurements. Secondly, it provides instantaneous alerts when a pre-set sound threshold (in decibels) is exceeded. Thirdly, it is capable of functioning in noisy environments, such as classrooms. Finally, the application is freely available on the Google Play Store. To adapt the wearable device (originally developed to measure noise) to measure vocal intensity, a collar microphone (Lsgoodcare, 3.5 mm plug, frequency response: 20 Hz–20,000 Hz) was connected to a Huawei P30 smartphone. The collar microphone was used to ensure that the recorded signal predominantly captured the participants’ voice rather than ambient environmental noise. This configuration was illustrated in Fig. 3. To mimic the real-world teaching situation, the Sound Meter application was not calibrated beforehand.

**Figure 4:**
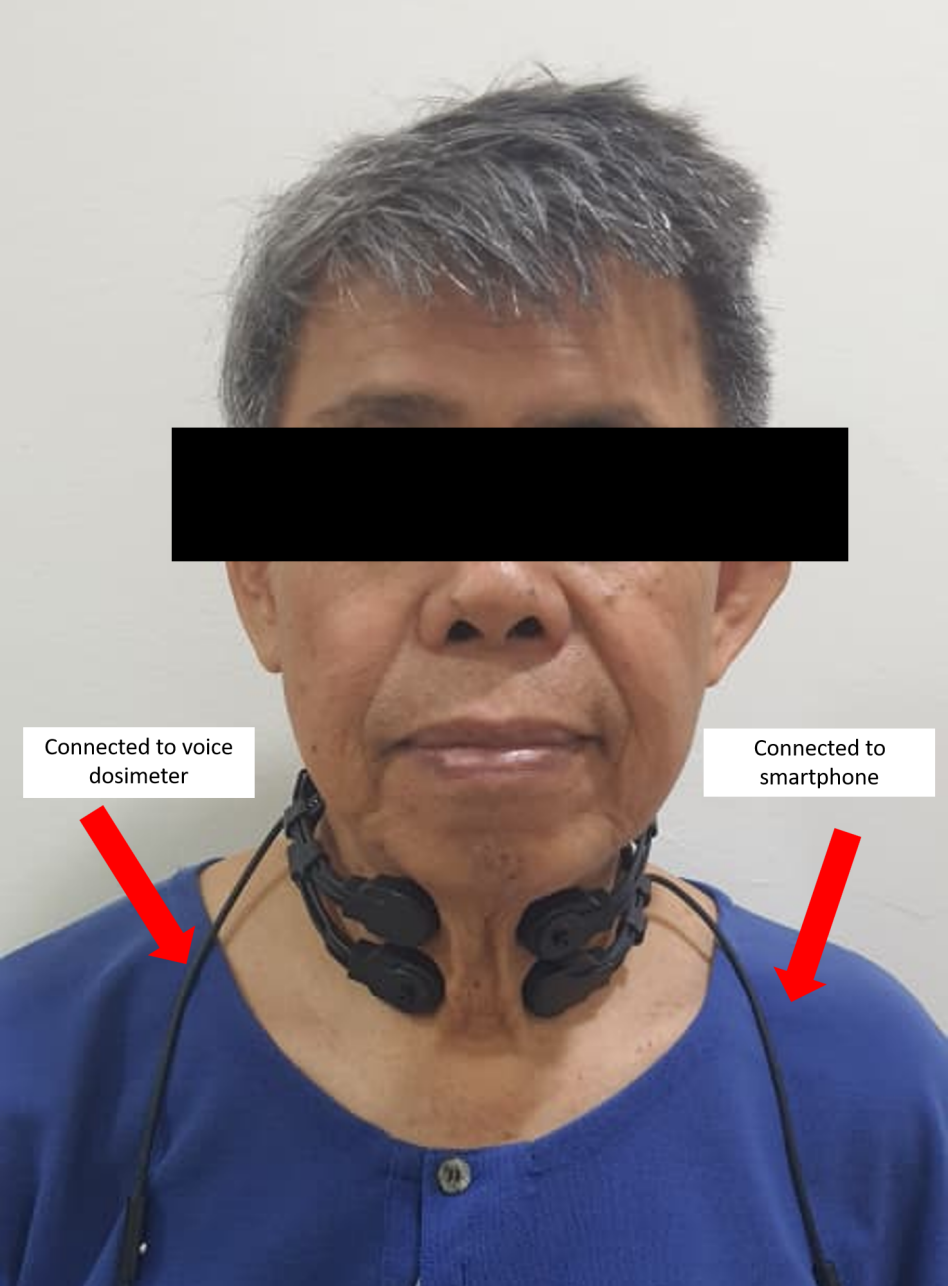
Position of the voice dosimeter and wearable device on the teacher.

During data collection, two sound-measuring devices, the wearable device and the voice dosimeter, were positioned as close as possible to the larynx, as illustrated in Fig. 4. Participants were instructed to attach both the wearable device microphone and the voice dosimeter to their necks. Both devices measured participants’ vocal intensity simultaneously. To ensure that only the participants’ vocal intensity was recorded and not environmental noise (*e.g.*, students shouting, fan noise, *etc.*), participants were instructed to place both devices underneath their clothing (as appropriate). They were also required to wear both devices for an unscripted class session (approximately 30 min). Participants were reminded that both devices measured only vocal intensity (*i.e.,* L_eq_) and not their speech content. They were instructed to conduct their lessons as usual without any scripted modifications to ensure ecological validity of vocal use.

In this study, vocal intensity was quantified in terms of sound pressure level (in decibels). This parameter was measured by both the wearable device and the voice dosimeter. Prior to data collection, all researchers underwent a short briefing on how to handle the wearable device and the voice dosimeter.

Descriptive analyses were conducted to analyse the sound pressure level as measured by both the wearable device and the voice dosimeter. Validation of the wearable device was evaluated using a concurrent validity test against the voice dosimeter (gold standard). Concurrent validity was assessed by calculating the correlation between the sound pressure level measured by the wearable device and that measured by the voice dosimeter. Pearson correlation was used to assess concurrent validity. A Pearson’s correlation coefficient (r) between 0.7 and 1.0 indicated a strong correlation between the sound pressure level as measured by the two devices, an r between 0.4 and 0.7 indicated a moderate correlation, and an r less than 0.4 indicated a weak correlation (Schober, Boer & Schwarte, 2018). Concurrent validity of the wearable device was considered acceptable if the Pearson correlation coefficient was at least 0.50 (Schober, Boer & Schwarte, 2018).

The intraclass correlation coefficient (ICC) was computed using a two-way mixed-effects model with absolute agreement [ICC (3,1)] to evaluate the inter-rater reliability of the sound pressure level as measured by the wearable device and the voice dosimeter (gold standard). An ICC greater than 0.90 indicated excellent reliability, an ICC between 0.75 and 0.90 indicated good reliability, an ICC between 0.50 and 0.75 indicated moderate reliability, and an ICC below 0.50 indicated poor reliability (Han, 2020). In this study, an ICC of at least 0.50 was set as the minimum standard for acceptable inter-rater reliability (Han, 2020). A significance level of 0.05 was used for all statistical analyses. All statistical analyses were conducted using SPSS Version 28 while acoustic analysis was performed *via* Praat version 6.4.48.

### Ethical approval

Ethical approval for this study was obtained from the National University of Malaysia Research Ethics Committee (JEP-2024-903), the Ministry of Education, Malaysia (KPM.600-3/2/3-eras(22830)), and the Negeri Sembilan State Education Office (JPPNS.500-8/9/1Jld2(18). An information booklet outlining the objectives and procedures of the study was provided to the management of the Ministry of Education Malaysia, the Negeri Sembilan State Education Office, and the participating schools. Only sound pressure level data were recorded, and no speech content was captured. Written informed consent was obtained from all teachers prior to their participation, covering in-class measurements, and all identifiable information was removed from the dataset. Participants received a RM10 voucher as appreciation for their involvement. All data collection and analysis adhered to the principles outlined in the Declaration of Helsinki.

## Results

A total of 132 participants (response rate = 92.3%) were recruited, with a mean age of 42.7 ± 0.8 years (Table 2). The median teaching hours per week of these participants was 17.5 (IQR: 14.0–28.0) hours. A majority of the participants were female (87.9%), of Malay ethnicity (64.4%), and employed at national primary school (66.7%). Of note, most participants reported using a voice amplifier while teaching (62.9%).

**Table 2:** Sociodemographic characteristics of the participants (*N* = 132).

**Variables**	**Mean (SD)**	**Median (IQR)**	**Frequency (%)**
Age (years)	42.7 (0.8)	–	–
Teaching Hours Per Week (hours)	–	17.5 (14.0–28.0)	–
Gender			
Male	–	–	16 (12.1)
Female	–	–	116 (87.9)
Ethnicities			
Malay	–	–	85 (64.4)
Chinese	–	–	26 (19.7)
Indian	–	–	18 (13.6)
Others	–	–	3 (2.3)
Types of School			
National Primary School	–	–	88 (66.7)
Chinese Primary School	–	–	29 (22.0)
Tamil Primary School	–	–	15 (11.4)
Use Voice Amplifier			
Yes	–	–	49 (31.7)
No	–	–	83 (62.9)

Overall, the total M-VHI-10 score was 17.0 (IQR: 12.0–21.8). Teachers in Chinese primary schools recorded the highest scores [19.0 (IQR: 14.0–28.0)], followed by those in national primary schools [17.0 (IQR: 12.0–20.0)], and Tamil primary schools [14.0 (IQR: 10.0–26.0)]. Nonetheless, the differences in M-VHI-10 scores across school types were not statistically significant (*p* = 0.214). These findings were tabulated in Table 3.

**Table 3:** M-VHI-10 scores according to the types of school.

**No. of participants from each type of school**	**Functional handicap,** **Median (IQR)**	**Physical handicap, Median (IQR)**	**Emotion handicap, Median (IQR)**	**Total score, Median (IQR)**	**Kruskal–Wallis**	***P*-value**
National Primary School (*n* = 88)	9.0 (6.0–10.8)	4.0 (3.0–4.0)	4.0 (3.0–6.0)	17.0 (12.0–20.0)	3.09	0.214
Chinese Primary School (*n* = 29)	10.0 (7.0–14.0)	5.0 (3.0–5.5)	4.0 (3.0–7.0)	19.0 (14.0–28.0)		
Tamil Primary School (*n* = 15)	7.0 (5.0–14.0)	4.0 (2.0–5.0)	3.0 (3.0–6.0)	14.0 (10.0–26.0)		
Total (*N* = 132)	9.0 (6.0–12.0)	4.0 (3.0–5.0)	4.0 (3.0–6.0)	17.0 (12.0–21.8)		

The overall prevalence of vocal handicap was 79.5% (95% CI [71.7–86.1]%) when a cut-off score of >11 was applied. However, using a more stringent cut-off of >7.5 points, all teachers in this study were classified as having vocal handicap. When analysed by severity, it was found that most of the teachers experienced mild vocal handicap (63.8%, 95% CI [53.9–73.0]%). Only a minority of the teachers had moderate (23.8%, 95% CI [16.0–33.1]%) and severe vocal handicap (12.4%, 95% CI [6.8–20.2]%). When analysed by school type, the highest prevalence occurred among teachers in Chinese primary schools (86.2%, 95% CI [68.3–96.1]%), followed by those in national primary schools (81.8%, 95% CI [72.2–89.2]), and Tamil primary schools (53.3%, 95% CI [26.6–78.7]%). This difference was significant (*X*^2^ = 7.40, *df* = 2, *p* = 0.025) (Table 4).

**Table 4:** Prevalence of vocal handicap according to types of school.

**No. of participants from each type of school**	**Frequencies of vocal handicap**	Prevalence of vocal handicap, 95% CI (%)^*^	**Chi-Square**	**df**	***P*-value**
National Primary School (*n* = 88)	72	81.8 (72.2–89.2)	7.40	2	0.025
Chinese Primary School (*n* = 29)	25	86.2 (68.3–96.1)			
Tamil Primary School (*n* = 15)	8	53.3 (26.6–78.7)			
Total (*N* = 132)	105	79.5 (71.7–86.1)			

**Notes.**

* A cut-off score of >11 points was used in the calculation of prevalence.

When examined by voice amplifier use, teachers who used a voice amplifier showed a higher prevalence of vocal handicap (89.8%, 95% CI [77.8–96.6]%) compared with those who did not (73.5%, 95% CI [62.7–82.6]%). These findings were presented in Table 5. This difference was also significant (*X*^2^ = 5.03, *df* = 1, *p* = 0.025).

**Table 5:** Prevalence of vocal handicap according to the status of vocal amplifier usage.

**Participants who use voice amplifier**	**Frequencies of vocal handicap**	Prevalence of vocal handicap, 95% CI (%)^*^	**Chi-Square**	**df**	***P*-value**
Yes (*n* = 49)	44	89.8 (77.8–96.6)	5.03	1	0.025
No (*n* = 83)	61	73.5 (62.7–82.6)			
Total (*N* = 132)	105	79.5 (71.7–86.1)			

**Notes.**

* A cut-off score of >11 points was used in the calculation of prevalence.

The mean sound pressure level recorded by the wearable device was 51.85 ± 2.27 dB, whereas the voice dosimeter recorded a higher mean level of 59.04 ± 3.03 dB, as presented in Table 6.

**Table 6:** The mean sound pressure level as measured by wearable device and voice dosimeter.

**Devices**	**Sound pressure level (in dB), Mean ± SD**
Wearable Device	51.85 ± 2.27
Voice Dosimeter	59.04 ± 3.03

The sound pressure level measured by the wearable device showed a weak correlation with that obtained from the voice dosimeter (*r* = 0.184, *p* = 0.330), indicating poor concurrent validity of the wearable device. These findings were presented in Table 7.

**Table 7:** Pearson’s correlation as concurrent validity test.

**Parameter**	**Pearson’s Correlation, r**	**95% CI**	***P*-value**
Sound Pressure Level (dB)	0.184	−0.189–0.510	0.330

The intraclass correlation (ICC [3,1]) for sound pressure level between the wearable device and the voice dosimeter was 0.077 (*p* = 0.171), indicating poor agreement; consequently, the wearable device demonstrated poor inter-rater reliability. These results were shown in Table 8.

**Table 8:** Inter-rater intra-class correlation (3, 1) as reliability test.

**Parameter**	**ICC (3, 1)**	**95% CI**	***P*-value**
Sound Pressure Level (dB)	0.077	−0.109–0.322	0.171

**Figure 5:**
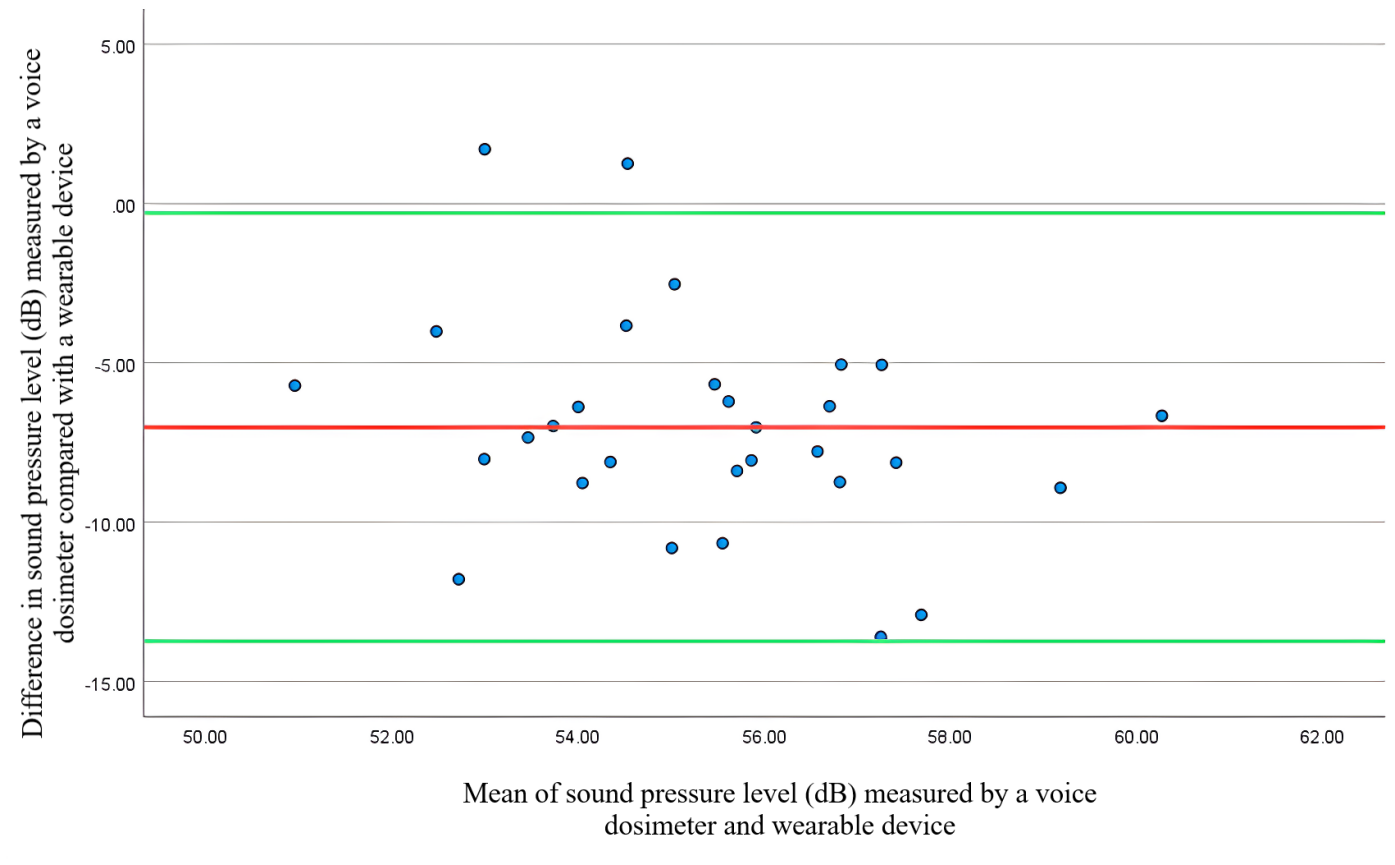
A Bland-Altman plot.

A Bland-Altman analysis was conducted (Fig. 5). It was found that the mean difference was −7.02, indicating that the wearable device tended to underestimate measurements compared with the voice dosimeter. The limits of agreement ranged from −13.75 to −0.29. Although most observations fell within these limits, the range exceeded the predefined clinically acceptable threshold of 5 dB, suggesting poor agreement between the two methods. Only two points lay outside the limits of agreement, indicating minimal outliers. No evidence of proportional bias was observed.

## Discussion

To the best of our knowledge, this is the first study that investigated the prevalence of vocal handicap among primary school teachers in Malaysia. Furthermore, this is the first study to validate a wearable device to measure vocal intensity against a voice dosimeter (as gold standard).

In the present study, the prevalence of vocal handicap was 79.5%, using the original VHI-10 cut-off score (>11). Although a lower cut-off of 7.5 points has been proposed among the Malaysian general population (Idris et al., 2025), adapting such a cut-off score will result in universal classification of vocal handicap (*i.e.,* 100% of the participants have vocal handicap). This could be due to the fact that teachers have greater vocal demand than the general population. As a result, the 7.5-cut-off value may not be universally adaptable to our teacher population and highlights the need for further validation of the M-VHI-10 threshold in this specific occupational group.

The prevalence of vocal handicap among primary school teachers in Malaysia appears to be higher than that reported in other countries, which ranged from 20.2% to 55.5% (Sampaio et al., 2012; Melo et al., 2023; Alharbi et al., 2024). This observation could be due to the fact that classrooms in public primary schools in Malaysia are often larger, noisier, and more crowded compared to other countries (Busst, 2024). Hence, teachers in Malaysia have to raise their voices frequently to overcome that substantial background noise from the students. Another plausible explanation is that teachers in Malaysian primary schools have limited awareness and training in vocal health, vocal hygiene, voice conservation techniques, which allow for early recognition of voice problems (Ang & How, 2021). As a result, harmful vocal behaviours such as shouting, inadequate hydration, and insufficient voice rest may persist, increasing the prevalence of vocal handicap over time. In addition, the prevalence in the study was also higher than that among secondary school teachers in Malaysia (Moy et al., 2015). This can be explained by the fact that primary school teaching inherently involves greater vocal demands, as younger children require more frequent instructions, repetition, storytelling, singing, and behavioural control. These activities often necessitate sustained voice use at higher intensities, increasing vocal load and the risk of vocal strain compared to secondary school teaching, where students are generally more independent and classroom management requires less vocal effort. Furthermore, with higher teaching workloads (Munier & Farrell, 2015) but limited opportunities for vocal rest in primary school, teachers may have to continue teaching despite significant vocal handicap.

The higher prevalence of vocal handicap among teachers who use voice amplifiers may initially seem counterintuitive, but several explanations can clarify this observation. For instance, voice amplifier users may be more likely to teach larger or noisier classes, spend longer hours teaching, or have fewer breaks, all of which increase the risk of vocal handicap. Another explanation is that teachers who use voice amplifiers may be more aware of their voice health and more likely to report symptoms (Banks, Cantor-Cutiva & Hunter, 2025), whereas non-users may underreport their voice issues. That said, a reverse causation is also possible, whereby teachers who already experience vocal handicap may be more likely to adopt voice amplifiers to reduce strain (Bovo et al., 2013; Gaskill, O’Brien & Tinter, 2012). In other words, the presence of vocal handicap may lead to voice amplifier use, rather than the device causing voice problems. This is particularly relevant in the cross-sectional design used in this study, in which data are collected at a single time point and causal relationships cannot be established. Regardless of the causality, the higher prevalence among voice amplifier users does not imply that amplifiers are harmful. Instead, it underscores that voice amplifiers are often adopted reactively and without integration into comprehensive voice care strategies. In many cases, teachers may begin using voice amplifiers only after experiencing significant voice issues, meaning that the devices are adopted reactively rather than preventively. Without complementary voice care practices, such as maintaining hydration, practicing proper vocal technique, and allowing sufficient voice rest, voice amplifiers alone may not prevent further vocal strain (Bovo et al., 2013).

Of note, the highest prevalence of vocal handicap was observed among teachers in Chinese primary schools, followed by National and Tamil primary schools. This finding may be explained by several factors. Firstly, teachers in Chinese primary schools may experience larger class sizes, which increase the overall vocal demand during teaching (GogoKids, 2025). Secondly, the more intensive academic requirements and longer teaching hours may further contribute to prolonged voice use throughout the school day (Chai, 2025). Thirdly, staffing shortages and workload imbalances in some Chinese primary schools due to limited funding may also exacerbate the situation, where less optimal teacher-to-student ratios result in teachers needing to cover more subjects or take on additional classes (Lim, 2017). Collectively, these factors may lead to sustained vocal strain and increased risk of vocal handicap.

Although the wearable device uses a collar microphone similar to that of a voice dosimeter, it was found that such hardware adaptation still appears to fail to provide valid and reliable measurements of voice intensity. One possible limitation lies within the device’s electronics and signal processing. Even with similar microphone specifications and placement over the larynx, and both devices measured sound pressure level using A-weighting, the wearable device’s internal circuitry, analog-to-digital conversion, and software algorithms may be less sophisticated than those in a voice dosimeter (Llorente et al., 2024; Popolo et al., 2002). Lower sampling rates, signal smoothing, or simplified filtering can result in inaccurate representation of rapid fluctuations or peak voice intensities. Environmental factors in real-world teaching settings add another layer of complexity. Classrooms are noisy, reverberant, and full of competing sounds that may interfere with the wearable device’s ability to isolate the teacher’s voice. While voice dosimeters often incorporate noise-reduction algorithms or calibration procedures to account for ambient sound (Llorente et al., 2024), the lack of formal calibration of the wearable device may have introduced a systematic bias, as reflected by the mean difference of approximately 7 dB, indicating consistent underestimation relative to the voice dosimeter.

### Strengths and limitations

A key strength of this study is its novelty, as it is the first to report the prevalence of vocal handicap among primary school teachers in Malaysia. Second, this study is the first to validate a wearable device for measuring vocal intensity against a voice dosimeter, the accepted gold standard. Third, voice intensity was measured in actual teaching environments, instead of within an acoustic laboratory. This enhances the ecological validity and relevance of the findings to everyday classroom conditions.

Nevertheless, several limitations should be acknowledged. First, the wearable device assessed voice intensity solely on sound pressure level. Other acoustic parameters that may influence vocal handicap, including fundamental frequency (F_0_), jitter, shimmer, harmonics-to-noise ratio (HNR), and vocal dose, were not evaluated. This limitation arises because sound pressure level was the only parameter that could be measured by both the wearable device and the voice dosimeter. Second, the wearable device remains susceptible to subtle environmental sounds, such as those from fans or passing vehicles, which may have compromised measurement reliability. In addition, it was not calibrated prior to usage (to mimic real-life practice among teachers). Third, the exclusion of teachers with self-reported or diagnosed voice disorders during the study period (in order to reduce potential confounding in the Phase 2 validation stage) may have introduced selection bias, causing the prevalence of vocal handicap among teachers to be underestimated. Finally, the risk factors for vocal handicap were not examined in this study. To better understand the higher prevalence of vocal handicap (than other countries) and to identify high-risk groups for targeted interventions, it was ideal to investigate these risk factors. However, exploring risk factors was beyond the scope and objectives of the present study.

### Recommendations for future studies

Future research should validate the wearable device beyond sound pressure level to include additional acoustic parameters such as fundamental frequency (F_0_), jitter, shimmer, HNR, and vocal dose. Evaluating these parameters would provide a more comprehensive and fairer comparison between wearable device and voice dosimeter. In addition, examining individual, occupational, and environmental risk factors for vocal handicap is important to identify high-risk groups and guide targeted interventions.

To develop a valid and reliable wearable device for measuring voice intensity, future studies could explore existing mobile applications specifically designed for voice monitoring, featuring enhanced noise filtering and greater sensitivity to soft-tissue transmission to improve accuracy and reliability in real-world conditions. If suitable applications are not available, collaboration with information technology experts would be necessary to develop such a voice-measuring application.

Additionally, strategies to account for anatomical variability, such as adjustable microphone fittings, standardised placement protocols, or calibration algorithms, would further improve measurement consistency across participants. Future studies including teachers with active voice disorders would provide a more comprehensive estimate of prevalence. Finally, increasing sample diversity and size by including teachers from different regions, school types, and age groups would enhance generalisability and provide insight into demographic and environmental influences on vocal health.

### Implications of the current study

In terms of practice, the higher prevalence of vocal handicap among primary school teachers, compared with other countries, underscores the importance of raising awareness about occupational vocal health in Malaysia. Teachers are encouraged to self-monitor their vocal hygiene by using simple tools such as the M-VHI-10.

In terms of research, while wearable devices are often adapted from technologies originally developed for environmental noise measurement, their performance in capturing voice-specific parameters may be influenced by methodological and contextual factors. Therefore, the observed limitations do not necessarily reflect the inherent capability of wearable technologies in general, but rather the implementation and calibration approach used in this study. Future improvements could enhance system performance and measurement validity. These include ensuring stricter alignment of acoustic weighting, implementing more rigorous calibration procedures at defined reference levels. In addition, controlled bench testing under standardised acoustic conditions prior to field deployment may help to isolate device-related measurement error and improve agreement with reference instruments.

Finally, the findings have important implications for workplace health policies. The Ministry of Education Malaysia and school management should consider developing guidelines and interventions aimed at enhancing vocal hygiene programmes, adjusting teaching workloads, and implementing other occupational health measures to safeguard the vocal health of primary school teachers.

## Conclusion

The prevalence of vocal handicap among primary school teachers in Negeri Sembilan, Malaysia was found to be higher than other countries. While it was initially intended to act as a surrogate tool for measuring vocal intensity to help prevent vocal handicap, the wearable device used in this study, which was originally designed for noise measurement, demonstrated poor validity and reliability for measuring voice intensity. Therefore, there is a need to develop a novel device specifically designed for accurate vocal intensity measurement.

##  Supplemental Information

10.7717/peerj.21522/supp-1 Supplemental Information 1 Prevalence of Vocal Handicap

10.7717/peerj.21522/supp-2 Supplemental Information 2 Validation Data

10.7717/peerj.21522/supp-3 Supplemental Information 3 Coding Book

10.7717/peerj.21522/supp-4 Supplemental Information 4 STROBE checklist

## Additional Information and Declarations

### Field Study Permissions

The following information was supplied relating to field study approvals (*i.e.*, approving body and any reference numbers): Ministry of Education, Malaysia.

### Data Availability

The following information was supplied regarding data availability: The data is available in the Supplemental Files.

## References

[ref-1] Adamu A, Jibril YN, Kolo ES, Aluko AA, Bello-Muhammad N, Ajiya A, Shuaibu IY (2022b). Evaluation of voice handicap and emotional status among dysphonic and non-dysphonic individuals: a cross-sectional survey. Amrita Journal of Medicine.

[ref-2] Alharbi NS, Alotaibi S, Alnughaythir AI, Abohelaibah F, Alruways AQ, Alharbi R, Alzahrani SA, Alsaedi H, Alotaibi B, Alharbi NS, Alotaibi S, Alnughaythir AI, Abohelaibah F, Alruways A, Alharbi R, Alzahrani SA, Alsaedi H, Alotaibi B (2024). Prevalence and risk factors of voice disorders among teachers in Saudi Arabia. Cureus.

[ref-3] Aliverti A (2017). Wearable technology: role in respiratory health and disease. Breathe.

[ref-4] Almuzaini HI (2025). Voice problems in school teachers: a cross-sectional study from Madinah, Saudi Arabia. Noise Health.

[ref-5] Ang M-F, How DS (2021). A vocal health survey among primary school teachers in Klang Valley, Malaysia. International Journal of Academic Research in Business and Social Sciences.

[ref-6] Baghban K, Golmohammadi G, Asadollahpour F (2025). The worldwide prevalence of voice disorders among schoolteachers: a systematic review and meta-analysis. Journal of Voice.

[ref-7] Banks RE, Cantor-Cutiva LC, Hunter E (2025). Factors influencing teachers’ experience of vocal fatigue and classroom voice amplification. Journal of Voice.

[ref-8] Bottalico P, Nudelman CJ (2023). Do-it-yourself voice dosimeter device: a tutorial and performance results. Journal of Speech, Language, and Hearing Research.

[ref-9] Bovo R, Trevisi P, Emanuelli E, Martini A (2013). Voice amplification for primary school teachers with voice disorders: a randomized clinical trial. International Journal of Occupational Medicine and Environmental Health.

[ref-10] Busst N (2024). Overcrowding, inconsistencies driving Bumi kids to international schools. Free Malaysia Today — FMT.

[ref-11] Castillo-Allendes A, Cantor-Cutiva LC, Vidal V, Hunter EJ (2024). Voice as a working tool for teachers: a qualitative study of work-related perceptions and impact. Journal of Voice.

[ref-12] Chai J (2025). Malaysia’s chinese primary schools: saved yet threatened by rising inflows of Malay students. Fulcrum. 2025/05/30/T08:00:00+00:00.

[ref-13] Feng S, Weng C, Cai S, Yang Z, Wu M, Kang N (2022). The prevalence and risk factors for perceived voice disorders in public school teachers. Laryngoscope Investigative Otolaryngology.

[ref-14] Fujiki RB, Huber JE, Sivasankar MP (2021). Restoration strategies following short-term vocal exertion in healthy young adults. Journal of Speech, Language, and Hearing Research.

[ref-15] Gaskill CS, O’Brien SG, Tinter SR (2012). The effect of voice amplification on occupational vocal dose in elementary school teachers. Journal of Voice.

[ref-16] GogoKids (2025). SJK(C) *vs.* SK schools: 10 key differences every parent should know. https://www.gogokids.my/articles/primary-school-sjkc-vs-sk-schools-10-key-differences-every-parent-should-knows.

[ref-17] González AD, De Almeida Lopes ACB, De Andrade SM, Gabani FL, Da Silva Santos MC, Rodrigues R, Mesas AE (2022). Schoolteachers with voice handicap are twice as likely to report depressive symptoms. European Archives of Oto-Rhino-Laryngology.

[ref-18] Han X (2020). On statistical measures for data quality evaluation. Journal of Geographic Information System.

[ref-19] Heller-Stark A, Maxfield L, Herrick J, Smith M, Titze I (2024). Comparative study of two semi-occluded vocal tract protocols: a randomized clinical trial. Journal of Speech, Language, and Hearing Research.

[ref-20] Hughes A, Shandhi MMH, Master H, Dunn J, Brittain E (2023). Wearable devices in cardiovascular medicine. Circulation Research.

[ref-21] Hunter EJ, Cantor-Cutiva LC, Van Leer E, Van Mersbergen M, Nanjundeswaran CD, Bottalico P, Sandage MJ, Whitling S (2020). Toward a consensus description of vocal effort, vocal load, vocal loading, and vocal fatigue. Journal of Speech, Language, and Hearing Research.

[ref-22] Idris F, Azman M, Mohd Zawawi NS, Mat Baki M (2025). Determining cutoff point in Bahasa Malaysia version of voice handicap index-10 (mVHI-10). Journal of Voice.

[ref-23] Joseph BE, Joseph AM, Jacob TM (2020). Vocal fatigue-do young speech-language pathologists practice what they preach?. Journal of Voice.

[ref-24] Kish L (1995). Survey sampling.

[ref-25] Kong F, Zou Y, Li Z, Deng Y (2024). Advances in portable and wearable acoustic sensing devices for human health monitoring. Sensors.

[ref-26] Lim I (2017). What you should know about Chinese schools in Malaysia. Malay Mail. 2017 2017/07/03/06:57:36.

[ref-27] Llorente M, Podhorski A, Fernandez S, Llorente M, Podhorski A, Fernandez S (2024). Wearable voice dosimetry system. Applied Sciences.

[ref-28] Melo LdS, Santos VM, Rocha JSB, Silva RRV, Haikal DSA, Rossi-Barbosa LAR, Souza LR, Medeiros AMd (2023). Vocal handicap and association with physical inactivity and job dissatisfaction among teachers. PsychTech & Health Journal.

[ref-29] Misono S, Banks K, Gaillard P, Goding GS, Yueh B (2015). The clinical utility of vocal dosimetry for assessing voice rest. Laryngoscope.

[ref-30] Moghtader M, Soltani M, Mehravar M, JafarShaterzadehYazdi M, Dastoorpoor M, Moradi N (2020). The relationship between vocal fatigue index and voice handicap index in university professors with and without voice complaint. Journal of Voice.

[ref-31] Moreno M, Calvache C, Cantor-Cutiva LC (2025). Systematic review of literature on prevalence of vocal fatigue among teachers. Journal of Voice.

[ref-32] Moy FM, Hoe VCW, Hairi NN, Chu AHY, Bulgiba A, Koh D (2015). Determinants and effects of voice disorders among secondary school teachers in peninsular Malaysia using a validated Malay version of VHI-10. PLOS ONE.

[ref-33] Munier C, Farrell R (2015). Working conditions and workplace barriers to vocal health in primary school teachers (the relationship between working conditions and voice in primary school teachers). Journal of Voice.

[ref-34] Ong FM, Husna Nik Hassan NF, Azman M, Sani A, Mat Baki M (2019). Validity and reliability study of Bahasa Malaysia version of voice handicap index-10. Journal of Voice.

[ref-35] Phyland D, Miles A (2019). Occupational voice is a work in progress: active risk management, habilitation and rehabilitation. Current Opinion in Otolaryngology & Head and Neck Surgery.

[ref-36] Popolo P, Rogge-Miller K, Svec J, Titze I, Gould W (2002). Technical considerations in the design of a wearable voice dosimeter. NCVS Online Technical Memo.

[ref-37] Reddy KD, Chawla S (2025). Wearable technology in gastroenterology: current applications and future directions. Journal of Clinical Medicine.

[ref-38] Sampaio MC, Borges dos Reis EJF, Carvalho FM, Porto LA, Araújo TM (2012). Vocal effort and voice handicap among teachers. Journal of Voice.

[ref-39] Schober P, Boer C, Schwarte LA (2018). Correlation coefficients: appropriate use and interpretation. Anesthesia & Analgesia..

[ref-40] Shoeib RM, Nassar JF, Ghandour HH (2012). Anxiety in female teachers with dysphonia: correlation between the voice handicap index and anxiety state. The Egyptian Journal of Otolaryngology.

[ref-41] Singhal A, Chopra A (2021). Understanding the wearable technology.

[ref-42] Sundram ER, Norsa’adah B, Mohamad H, Moy FM, Husain NRN, Shafei MN (2019). The effectiveness of a voice care program among primary school teachers in northeastern Malaysia. Oman Medical Journal.

[ref-43] Titze IR, Švec JG, Popolo PS (2003). Vocal dose measures: quantifying accumulated vibration exposure in vocal fold tissues. Journal of Speech, Language, and Hearing Research.

[ref-44] Walter SD, Eliasziw M, Donner A (1998). Sample size and optimal designs for reliability studies. Statistics in Medicine.

[ref-45] Won SJ, Kim RB, Kim JP, Park JJ, Kwon MS, Woo SH (2016). The prevalence and factors associate with vocal nodules in general population. Medicine.

