# Supplementary material for: Prevalence of vocal handicap among primary school teachers in Malaysia and validation of a noise-measuring wearable device to measure vocal intensity
Source: PeerJ. 2026 Aug 13;14:e21522. doi: 10.7717/peerj.21522 (PMC13477642; doi:10.7717/peerj.21522)
Supplement: Supplementary file 1 [file peerj-14-21522-s003.docx]

**Coding for Categorical Data**

| **Categorical Variables** | **Names in Full** | **Coding** |
| --- | --- | --- |
| Gender | Gender | 1 = Male  2 = Female |
| Ethnicity |  | 1 = Malay  2 = Chinese  3 = Indian  4 = Others |
| School_Type | Types of school | 1 = Malay primary school  2 = Chinese primary school  3 = Tamil primary school |
| Use_Mic | Use of amplifier | 1 = Yes  0 = No |
